# Spatiotemporal Variation in Environmental *Vibrio cholerae* in an Estuary in Southern Coastal Ecuador

**DOI:** 10.3390/ijerph15030486

**Published:** 2018-03-10

**Authors:** Sadie J. Ryan, Anna M. Stewart-Ibarra, Eunice Ordóñez-Enireb, Winnie Chu, Julia L. Finkelstein, Christine A. King, Luis E. Escobar, Christina Lupone, Froilan Heras, Erica Tauzer, Egan Waggoner, Tyler G. James, Washington B. Cárdenas, Mark Polhemus

**Affiliations:** 1 Quantitative Disease Ecology and Conservation Lab, Department of Geography, University of Florida, Gainesville, FL 32610 USA; tjames95@ufl.edu; 2 Emerging Pathogens Institute, University of Florida, Gainesville, FL 32610, USA; 3 Center for Global Health and Translational Science, SUNY Upstate Medical University, Syracuse, NY 13210, USA; stewarta@upstate.edu (A.M.S.-I.); escobar1@vt.edu (L.E.E.); luponec@upstate.edu (C.L.); fheras_est@utmachala.edu.ec (F.H.); erica.tauzer@gmail.com (E.T.); egan.waggoner@gmail.com (E.W.); polhemum@upstate.edu (M.P.); 4 Laboratorio para Investigaciones Biomédicas, FCV, Escuela Superior Politécnica del Litoral, Guayaquil 090101, Ecuador; eeordone@espol.edu.ec (E.O.-E.); wbcarden@espol.edu.ec (W.B.C.); 5 Division of Nutritional Sciences, Cornell University, Ithaca, NY 14850, USA; wchu3278@gmail.com (W.C); jfinkelstein@cornell.edu (J.L.F.); 6 Department of Microbiology and Immunology, SUNY Upstate Medical University, Syracuse, NY 13210, USA; KingCh@upstate.edu; 7 Department of Fish and Wildlife Conservation, Virginia Tech, Blacksburg, VA 24061, USA

**Keywords:** cholera, Ecuador, *Vibrio cholerae*, strains O1 and O139, *Vibrio*, temperature, spatial

## Abstract

Cholera emergence is strongly linked to local environmental and ecological context. The 1991–2004 pandemic emerged in Perú and spread north into Ecuador’s El Oro province, making this a key site for potential re-emergence. Machala, El Oro, is a port city of 250,000 inhabitants, near the Peruvian border. Many livelihoods depend on the estuarine system, from fishing for subsistence and trade, to domestic water use. In 2014, we conducted biweekly sampling for 10 months in five estuarine locations, across a gradient of human use, and ranging from inland to ocean. We measured water-specific environmental variables implicated in cholera growth and persistence: pH, temperature, salinity, and algal concentration, and evaluated samples in five months for pathogenic and non-pathogenic *Vibrio cholerae*, by polymerase chain reaction (PCR). We found environmental persistence of pandemic strains O1 and O139, but no evidence for toxigenic strains. *Vibrio cholerae* presence was coupled to algal and salinity concentration, and sites exhibited considerable seasonal and spatial heterogeneity. This study indicates that environmental conditions in Machala are optimal for cholera re-emergence, with risk peaking during September, and higher risk near urban periphery low-income communities. This highlights a need for surveillance of this coupled cholera–estuarine system to anticipate potential future cholera outbreaks.

## 1. Introduction

Cholera, a disease caused by the Gram-negative bacteria *Vibrio cholerae*, remains a severe global threat to public health and development efforts [1]. A 2015 study estimated the global burden at 2.86 million cases, with 95,000 deaths, and 1.3 billion people at risk of cholera infection [2]. An analysis of global cholera pandemics indicated that cholera outbreaks originate in coastal regions, often during flooding events, before spreading inland [3]. Studies suggest outbreaks of *V. cholerae* can be explained by oceanographic variables (e.g., sea surface temperature, pH, salinity) and phytoplankton blooms, indicating the potential to predict disease outbreaks [3,4]. Blue-green algae (BGA, cyanobacteria) has been described as a reservoir for environmental cholera [5], in part because its growth raises pH, encouraging *Vibrio* growth, but also as the major food source for zooplankton. The association between *V. cholerae* and zooplankton, wherein *Vibrio* are highly concentrated on their carapaces and internally, makes untreated water consumption in coupled zooplankton-phytoplankton hotspots a major potential point of infection [6]. Our previous work suggests that both predicted current and future coastal hotspots of cholera transmission are far larger than current epidemiological surveillance efforts can capture [7]. Indeed, the links between climate change and cholera brings the role of environmental *V. cholerae* persistence into sharp relief [8], highlighting the importance of local surveillance efforts in vulnerable areas.

Estuarine systems are a natural intersection of coastal oceanographic conditions and human use; as productive systems for fisheries, port locations for transport, and rich riparian soils, they are a highly-exposed interface. Coastal estuarine systems often represent subsistence-level dependence on the interface, in terms of artisanal fisheries, a higher likelihood of direct water use, and simply greater physical exposure by proximity. This renders them the most vulnerable of populations, most likely to be exposed to pathogens, and in the most flooding-prone areas in the world [9,10,11,12,13,14]. The causative agent of cholera, *V. cholerae*, originates from estuarine waters. Evidence for this includes phylogenetic information and the physiological requirements for growth and persistence [15,16]. *V. cholerae* is environmentally persistent in the Bay of Bengal (Bangladesh and India), along coastal areas in Latin America [6,17], and in riverine, estuarine, and coastal waters around the world [6]. Cholera epidemics have historically followed coastlines [18], and *V. cholerae* can be transmitted to humans via a wide range of marine organisms, including zooplankton, aquatic plants, shellfish, and fish [19]. 

Ecuador is a critical location to understand cholera and other climate and water-sensitive diseases due to its: (1) high potential for cholera outbreaks, and the high incidence of other climate-sensitive infectious diseases (e.g., leptospirosis, dengue); and (2) the strong influence of oceanographic conditions on local climate and flooding during El Niño events [9,10,14,20,21]. In January 1991, cholera re-emerged in Latin America after more than a century without cases [22]. In Ecuador, the 1991 cholera epidemic emerged in the south of the country from a small fishing village in El Oro Province, and it is suspected that a fisherman introduced the index case was traveling north from Perú [12]. From 1991 to 2004 over 90,000 cases of cholera were reported in Ecuador, with most cases from coastal provinces. El Oro and Guayas provinces, located in southern coastal Ecuador, collectively represented one of two disease epicenters in the country. Recent studies indicate a high risk of a second epidemic in Ecuador due to the presence of important risk factors including the growth of vulnerable urban populations, decreased investment in cholera surveillance and prevention programs, increased flood risk associated with climate change, and a street food culture that includes eating raw shellfish (ceviche) [23]. In addition, Guayaquil (Guayas Province), the largest city in Ecuador, has been identified as the third most vulnerable city in the world to future flood risk [9]. Furthermore, it has been found that in populations with a high prevalence of blood group O, such as in Latin America, illness from cholera is more severe, and the requirements for rehydration and hospitalization of infected individuals are considerably higher [24,25]. Given these conditions, there is compelling evidence that people in the southern coast of Ecuador are a high-risk population and there is a critical need for active cholera surveillance in this region. 

To address this, local variability in the presence of *V. cholerae* was evaluated in the estuarine environment surrounding the city of Machala, El Oro province, a site identified as a current and future coastal cholera hotspot [7]. Five sampling sites were selected, associated with estuarine water access in Machala, Ecuador, representing a range of economic and human activity conditions, in addition to different proximity to the ocean. Using water sampling methodology, coupled with laboratory identification of *V. cholerae* bacteria, the local environmental and pathogenic conditions were assessed for a period of ten months. Strengthening climate and water-sensitive infectious disease surveillance systems [26,27] and further understanding of the role of environmental factors in disease outbreak and transmission over time and space [28,29] are urgently needed to target cholera and other climate and water sensitive diseases. 

## 2. Materials and Methods

### 2.1. Study Site

**Figure 1:**
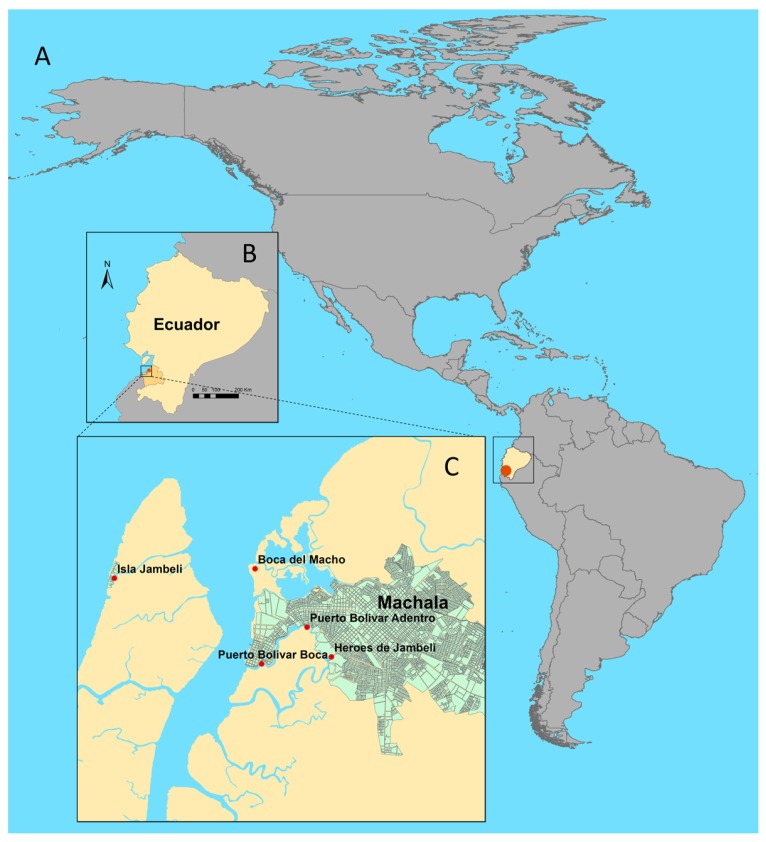
Location of sampling sites. (**A**) Ecuador (in yellow) in South America, indicating the location of Machala (red point); (**B**) the location of Machala on the southern coast of Ecuador (red point); and (**C**) the location of the five sampling sites: Isla Jambelí, Boca del Macho, Puerto Bolívar Boca, Puerto Bolívar Adentro, and Héroes de Jambelí (red points), in and around Machala (green).

Machala is a port city of approximately 250,000 inhabitants, with major economic activities stemming from agriculture (bananas), aquaculture (shrimp farming), and fishing/shellfish collection, both small-scale and semi-industrial scale. Five sampling sites (Isla Jambelí, Boca del Macho, Puerto Bolívar Boca, Puerto Bolívar Adentro, and Héroes de Jambelí) were established within the Machala estuarine system (Figure 1), selected for maximum heterogeneity, to include highly built urban areas, ports, mangrove, and coastal areas. Isla Jambelí is on the outer edge of the coastal draining estuary, and the entrance to Jambelí is interspersed with mangroves and shrimp farms. Boca del Macho is the open edge of the inner estuary, in open water on shallow sand, with mangroves. Puerto Bolívar Boca is near the mouth of the open harbor, characterized by heavy boat traffic, commercial fishing, and residences lining the waterway, with mangroves and shrimp farming on the far side of the waterway. Puerto Bolívar Adentro is within the city, on the estuary, in an area characterized by residential low-income housing, with shrimp farms and mangroves across the Héroes waterway. Héroes de Jambelí is the most inland site, characterized by low income and poor-quality housing built along mangroves at the edge of the city; outflow from the houses visibly drains directly into the water (Figure 1). The port city of Machala is an important sentinel surveillance site, due to its location along the Pan American highway, approximately 80 km north of the Peruvian border, facilitating significant movement of people and potential pathogens by land and sea.

### 2.2. Collection of Water Samples and Environmental Measurements

At each of the five study sites (Figure 1), water sampling was conducted at high tide, twice monthly along a transect with three sub-sites spaced 250 m apart, and three replicates per sub-site. Three 1 L surface water samples per sub-site were collected in sterile polypropylene bottles, and placed in coolers with ice for transport to the laboratory. Biological, chemical, and physical water characteristics were sampled using a YSI water probe* (600 XLM V2 Sonde, Yellow Springs Incorporated (YSI), Yellow Springs, OH, USA). Surface temperature (°C), conductivity, pH, salinity, and Optic-T BGA PE (Phycoerythrin) (blue-green algae) (cells/mL, which we converted to cells/µL for ease of visualization) were recorded, at each end of the transect.

### 2.3. Laboratory Analyses

Water samples were transferred to the laboratory in coolers for *V. cholerae* testing and were processed within 24 h of collection. For laboratory analysis, a 1 L water sample was filtered through a No. 1 Whatman membrane (11 μm pore size) and 0.22 μm membrane (Millipore, Darmstadt, Germany) by vacuum. Then, 10 mL of phosphate-buffered saline (PBS) (pH 7.4) was pipetted onto the retained contents on the membrane and gently washed by pipette 15x. The PBS was left on the membrane to incubate at room temperature for 15 min prior to collection in a 50 mL conical tube. Three milliliters of membrane-washed PBS was enriched in 27 mL alkaline peptone water (APW) (1% peptone, 1% NaCl, pH 8.6) and incubated for 24 h at room temperature. Ambient room temperature in the laboratory was recorded daily. Five milliliters of bacteria enriched with APW was then centrifuged at 4500 rpm for 10 min at 4 °C, the supernatant was decanted, and the pellet was frozen at −80 °C for DNA isolation.

### 2.4. DNA Isolation and PCR

Genomic DNA was extracted from bacterial pellet of the previous step with a QIAamp DNA mini kit (Qiagen, Hilden, Germany), following manufacture instructions. Multiplex PCR was used for identification of *Vibrio cholerae* serogroups and the detection of toxigenic genes. Table 1 describes the primer sets used to amplify the *rfb* (O antigen biosynthesis encoding) region of O1 and O139 serogroups and the toxin subunit A (ctxA) and toxin coregulated pilus (tcpA) genes. For both duplex PCRs, master mix was as follows: 0.05 U/uL of JumpStart REDTaq DNA Polymerase (Sigma, Darmstadt, Germany), 1X buffer, 0.2 mM dNTPs, 0.2 mM of each primer set, 1 µL of template and ultrapure water to a final volume of 25 µL. The amplification program for diagnosis of serogroups was adapted from Hoshino et al. [30] using the following conditions: 5 min at 94 °C, 35 cycles of 94 °C for 1 min, 55 °C for 1 min, and 72 °C for 1 min and final extension of 72 °C for 7 min. Positive samples for either or both serogroups were subjected to toxigenic genes duplex PCR. The amplification program was according to conditions described in Kumar et al. [31]: 3 min at 94 °C, 30 cycles of 94 °C for 30 s, 59 °C for 30 s, and 72 °C for 1.2 min, and final extension of 72 °C for 10 min. PCR products were resolved in a 2% agarose gel. Our positive extraction control was a strain of the *V. cholerae* O1 serogroup, kindly provided by the National Institute of Public Health Research (INSPI). As negative control we used an isolate of *Escherichia coli* DH5 alpha. Moreover, to validate our multiplex PCR methods, some positive O1 and O139 amplicons were cloned into a pGEM®-T plasmid vector (Promega, Madison, WI, USA) and then sent for sequencing to Genewiz Company (South Plainfield, NJ, USA). Sequences can be provided on request. 

### 2.5. Statistical Analyses

As the data were not normally distributed, we conducted non-parametric tests to explore differences between sites. Each water environmental variable: temperature, pH, salinity, and BGA, was examined using the Kruskal-Wallis rank sum tests on site means, and on monthly means. To explore the relationships between environmental variables and *V. cholerae* prevalence, and the separate strains (i.e., O1, O139), we used a generalized linear mixed model (GLMM), allowing us to control for site differences. We used the lme4 package in R [33], with prevalence specified as a ‘binomial’ distribution family, setting site (*n* = 5) as the random effect, and environmental variables (four variables, *n* = 25 observations) as fixed effects. 

## 3. Results

### 3.1. Water Sample Environmental Measurements

**Figure 2:**
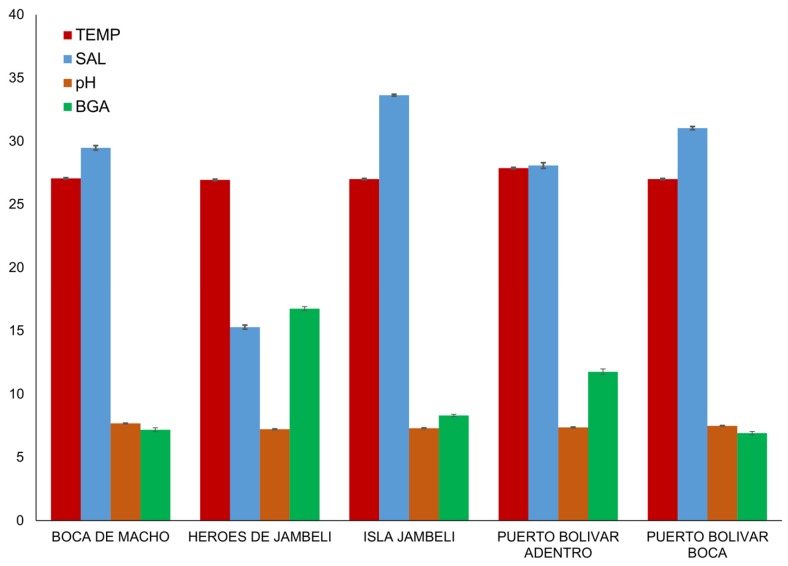
Water characteristics by site (means and standard errors temperature (TEMP, °C), salinity (SAL), pH, and measured total concentration of blue-green algae (BGA, cells/µL).

The probe recorded a range of 9–104 readings at each sub-site biweekly for 10 months. All readings were pooled to month for analyses. The sites differed significantly in environmental characteristics (Figure 2), as shown by a series of Kruskal-Wallis rank sum tests (temperature: χ^2^ = 206.19, df = 4, *p* < 0.0001; salinity: χ^2^ = 2257.5, df = 4, *p* < 0.0001; pH: χ^2^ = 1347.3, df = 4, *p* < 0.0001; BGA χ^2^ = 1824.8, df = 4, *p* < 0.0001). Héroes de Jambelí, the most inland site, had the highest recorded BGA, and Isla de Jambelí, the most coastal site, had the highest recorded salinity; while there were statistical differences between all sites in all characteristics, there were no clear outliers in pH or temperature. 

**Figure 3:**
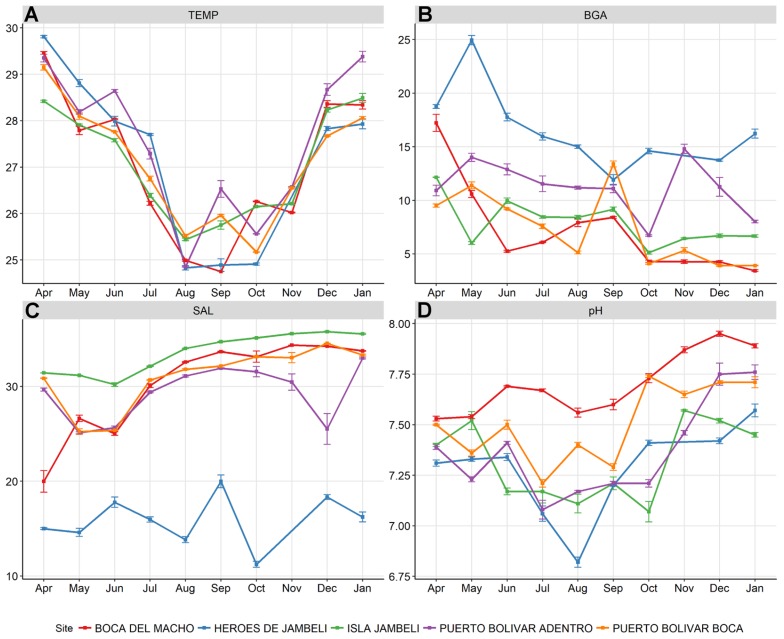
Environmental features in the study period. Water characteristics by month (means and standard errors) and sites: (**A**) temperature (TEMP, °C); (**B**) salinity (SAL); (**C**) measured total concentration of blue-green algae (BGA, cells/µL); and (**D**) pH.

The sites exhibited significant change in water characteristics across months (Figure 3), as shown by a series of Kruskal-Wallis rank sum tests (Table 2). Temperature was lowest in August for all sites—likely reflecting Pacific upwelling, which cools the water, regardless of air temperature. Salinity at the most inland site, Héroes de Jambelí, was consistently lowest, and showed the smallest change across months, while the other sites had a decrease in salinity in May, then a rise from July–December. Isla de Jambelí had the highest salinity, reflecting its location on the most coastal site. BGA was highest at the most inland site, Héroes de Jambelí, peaking in May, lagging temperature by a month. BGA shows the least temporal or spatial clustered pattern and has no obvious seasonality across the year. Héroes de Jambelí, however, registered the highest BGA values during the study (~25,000). pH appears to peak in December–January across all sites, with a decrease in July–August; the coastal and inland sites showed low pH values across seasons, while Boca del Macho registered consistently high pH values across months. 

### 3.2. Laboratory Analyses

**Figure 4:**
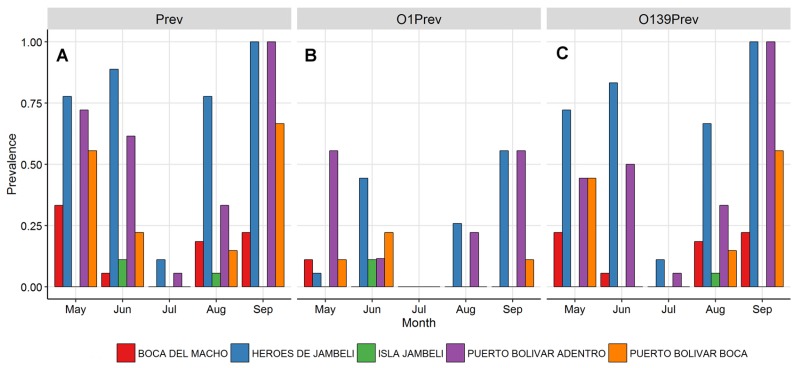
*Vibrio cholerae* detection. Monthly site prevalence of (**A**) *Vibrio cholerae* as given by positive PCR test; (**B**) O1 strain; and (**C**) O139 strain.

Of a total of 405 individual water samples, collected between May–September, 382 were diagnosed by multiplex PCR. There were 139 (36%) samples positive for *V. cholerae*, and 243 (64%) that were negative. Both O1 and O139 serogroups of *V. cholerae* were present in the estuarine system studied in Machala, Ecuador. Serogroup O139 was predominant; 118 (83.5%) samples were O139 and 51 (35.3%) were O1 (30 samples contained both). It was possible to detect *V. cholerae* during every one of the five months sampled; nevertheless, we found that prevalence decreased sharply in July (Figure 4). Positive and negative controls resulted as we expected. We confirmed the O1 positive strain provided by INSPI, however, we also found that this sample was positive for the ctxA toxigenic gene. By sequencing some randomly-chosen samples, it was confirmed that the PCR protocol applied was appropriate for the detection of *V. cholerae* serogroup O1 and O139 strains. Results from the toxin subunit A (ctxA) and toxin coregulated pilus (tcpA) analyses were all negative. There was no evidence found for persistent environmental toxigenic *V. cholerae* in these samples.

### 3.3. *Vibrio cholerae* Prevalence

Water samples were pooled within sites to derive monthly *V. cholerae* prevalences across and within sites (prevalence = positive/total samples tested). Overall monthly prevalence of *V. cholerae* ranged from 0.3 (*n* = 68) in July to 0.58 (*n* = 45) in September, with site prevalence ranging from 0 to 1, with a mean monthly site prevalence of 0.35 (Figure 4A). Individual strain prevalence was generally higher for O139 than O1, but Puerto Bolívar Adentro and Héroes de Jambelí were *V. cholerae* positive in every month, and also had higher prevalences than the other sites (Figure 4B,C). When controlling for site differences, we saw a significant association between the environmental variables and all *V. cholerae* prevalence, but associations only held for temperature, BGA (blue-green algae densities), and pH, for strain O139 alone, and none were significant for the O1 strain. We report fixed effect coefficient estimates, with Wald tests for significance (Table 3).

## 4. Discussion

Evidence for a persistent environmental reservoir of *V. cholerae* in the estuarine waters of Machala, Ecuador, in 2014 was found. The presence of *V. cholerae*, including pandemic strains O1 and O139 was confirmed. Ongoing toxigenic presence cannot be ruled out, but it was not detected in this study. Prior to 1961, epidemics of cholera were associated only with O1 strain, both Classic, and later, El Tor type, with the pathogenic O139 strain appearing in the 1992 pandemic in the Bay of Bengal, arising from genetic exchange with O1 El Tor [6]. Other pathogenic O1 strains are thought to have evolved and emerged independently, such as the U.S. Gulf Coast O1 strain, and the Australian clone [34], and there is evidence that toxigenic strains can arise from nontoxigenic environmental strains [35], including the toxigenic O139 strain. Without further investigation, the ongoing environmental *V. cholerae* presence in Machala cannot be attributed distinctly to a residual persistent reservoir since 1992, or repeated introduction of environmental strains from ballast water exchange at the port. However, there is persistence of the two strains most commonly associated with epidemics around the world. 

The sampling sites exhibited considerable seasonal and spatial heterogeneity in environmental characteristics and *V. cholerae* prevalence, with clear peaks (and troughs) during specific months. For example, there was peak *V. cholerae* prevalence in September, with highest values in two sites: Héroes de Jambelí and Puerto Bolívar Adentro (Figure 1). These sites are characterized by low-income housing on the edge of the city, while being inland sites, facing mangroves and shrimp farms. They were found to have *V. cholerae* present in every month sampled. The lowest *V. cholerae* prevalence occurred in July, in which only the two most inland sites had detectable *V. cholerae*. Water temperature had the clearest temporal pattern, falling rapidly through July, likely corresponding to Pacific upwelling, cooling the waters, and increasing nutrients in the system [36]. Unsurprisingly, the lowest salinity was recorded in the most inland site, Héroes de Jambelí, with a higher concentration of BGA than in other sites. This is in contrast to Isla Jambelí, a small island community furthest from the mainland and closest to the ocean, with high salinity due to its coastal location; however, it did not have significantly lower BGA than other sites. 

The timing of *V. cholerae* prevalence was coupled to the measured environmental water characteristics. For example, temperature, BGA, and pH decreased in most sites through July/August, as did the prevalence of *V. cholerae*, and statistically significant associations were found between prevalence and site- and month-specific temperature, salinity, pH, and BGA. However, we saw that these associations did not hold for both strains of *V. cholera*—specifically, none of the environmental variables were significantly associated with O1 strain alone, and salinity was no longer significantly associated with O139 strain prevalence when controlling for sites. Average ocean salinity is around 35 ppt, while freshwater rivers average around 0.5 ppt; this estuarine system is a mixed or brackish system, ranging from the lower average of around 15 ppt at the most inland site, to a high approaching 34 ppt at the coastal site. The most inland site represents optimal salinity for *V. cholerae* growth [37]. There was *V. cholerae* detected at a range of salinities, revealing an overall negative correlation with increasing salinity, indicating that lower salinity permits a suitable environment for the growth of *V. cholerae*. However, higher salinities approaching oceanic concentrations do not appear to completely prohibit growth. This finding is consistent with previous work demonstrating the suitability of coastal oceans for *V. cholerae* [36], but reveals a finer scale relationship with salinity in an estuarine system, up the gradient to fresh water. 

Blue-green algal density (BGA, also known as cyanobacteria), are photosynthetic prokaryotes found in freshwater, marine, and terrestrial environments [38]. The photosynthetic pigments of cyanobacteria include chlorophyll-*a* and the phycobiliproteins phycocyanin and phycoerythrin. Here BGA values were used to characterize water features, and because BGA has previously been associated with *V. cholerae* persistence [39]. Temperature increase, coupled with high nutrient load, low flow, and thermal stratification, generally results in increased growth rates of cyanobacteria, and its dominance in the phytoplankton community [40,41,42]. This could explain the high BGA values early in the year (Figure 3). In addition, warm temperatures promote increases in the number of days where BGA biomass exceeds warning thresholds established by WHO [40,43]. High temperature also influences water column stability and mixing depth, producing favorable conditions for BGA blooms [44,45]. This association of temperature increase with BGA blooms is consistent across coastal, estuarine, and inland waters [46], illustrating the suitability of tropical estuarine waters for environmental *V. cholerae* growth and persistence. Long-term monitoring to measure BGA biomass is recommended, and should be considered at a minimum in Héroes de Jambelí and Puerto Bolivar, the sites reporting the highest BGA values (Figure 2). This is particularly important, looking to the future, considering that a rise in water temperature—which we expect with global climate change—is associated with BGA emergence [47,48,49]. Given the strength of association between BGA and *V. cholerae* presence found in this study, compounded by the impact of temperature and pH, and a link to planktonic associations with environmental bacterial growth, this seems like a useful sentinel for surveillance. 

This study was conducted in an average climate year, providing a preliminary framework for monitoring coupled *V. cholera*—estuarine dynamics for potential emergence of cholera outbreaks in the region. This is a preliminary study, and with limited sample size, precluding larger conclusions or significant predictive power for the future. We note that this sample size may limit our capacity to fully elicit signals in our modeling approach, simply due to low number of *V. cholerae* findings (particularly O1 strain), within a highly variable system. Due to sample enrichment and multiplex PCR with specific primers sets, our procedure was sensitive to detecting cultivable *V. cholerae* from estuarine water samples. Nevertheless, for future studies we also suggest performing DNA isolation from viable but non-culturable *V. cholerae* (VBNC) directly from water samples. Moreover, we suggest that analysis of cholera incidence associated with plankton is also key to estimate the prevalence of the vibrio and its toxigenic genes. As the first study of this type in the region, and as a prototype for small-scale epidemic surveillance platforms, evidence is provided of the presence of these persistent environmental *V. cholerae* strains, and of the capacity to conduct such monitoring. This is particularly useful baseline information for anticipating El Niño years, extreme climate events associated with warming temperatures of surface ocean water, and increased rainfall and flooding events. In the year following the study, an El Niño year, there was severe urban flooding throughout Machala. Climate change projections indicate that the frequency of extreme El Niño events will increase in the future [14], increasing the risk of water-borne diseases endemic in the region, such as cholera, typhoid, and leptospirosis. This study provides valuable information, and our recommendation is to add *V. cholerae* back into the public health agenda, to consider infectious diseases beyond the already important vector-borne diseases, such as dengue fever, chikungunya, zika, and malaria. 

Indeed, in May 2016, two years after the initiation of this study, a case of cholera was reported in Machala, after approximately 12 years with no case reports in Ecuador [50]. An immuno-compromised individual was confirmed positive for *V. cholerae* serotype O1 non-toxigenic, by the National Public Health Research Institute of the Ministry of Health. Coauthors on this study diagnosed the patient using the same PCR assays described here, as the only lab in the region with this capacity. Although the source of the infection was not confirmed, this study report suggests a worrisome link to environmental transmission, underscoring the importance of our results.

## 5. Conclusions

This study highlights the urgency for active epidemiological and environmental waterborne pathogen surveys and the need for public health interventions to reduce the risk of water-borne pathogen transmission in this vulnerable population. The community Héroes de Jambelí is a low-income peri-urban settlement with less than 50 families, established informally in 2002. The community continues to lack adequate access to piped water, sewerage, and garbage collection due to their status as an illegal settlement. Simple bamboo homes have been built over the mangrove system, with direct discharge of wastewater into the estuary. At the same time, this community’s livelihood depends on artisanal fisheries (e.g., crabs, mollusks) from these same estuaries. Given the findings of environmental *V. cholerae* across the whole gradient of estuarine surface water, the full socio-economic spectrum of this port city is at risk. This vulnerable coupled human-natural system results in a high potential risk of emerging epidemics from water-borne pathogens. Furthermore, limited in sample size and duration, this study serves as a baseline from which to build, and draws attention to the gaps in surveillance in a region that has previously sourced pandemic cholera, and could again. 

## Figures and Tables

**Table 1:** PCR primers set used in this study.

Set	Primer	Sequence	Product	Reference
1	O1F2-1	GTT TCA CTG AAC AGA TGG G	192 bp	Hoshino et al. [30]
	O1R2-2	CGG TCA TCT GTA AGT ACA AC		
2	O139F2	AGC CTC TTT ATT ACG GGT GG	449 bp	
	O139R2	GTC AAA CCC GAT CGT AAA GG		
3	tcpA-F	ATG CAA TTA TTA AAA CAG CTT TTT AAG	675 bp	Kumar et al. [31]
	tcpA-R	TTA GCT GTT ACC AAA TGC AAC AG		
4	ctxA-F	CGG GCA GAT TCT AGA CCT CCT G	564 bp	Singh et al. [32]
	ctxA-R	CGA TGA TCT TGG AGC ATT CCC AC		

**Table 2:** Kruskal-Wallis rank sum tests for each site and environmental variable by month.

Environmental Variable	Site	χ^2^	DF	*p*-Value
Temperature	Boca de Macho	832.65	9	<0.0001
	Héroes de Jambelí	643.85	8	<0.0001
	Isla de Jambelí	622.85	9	<0.0001
	Puerto Bolívar Adentro	445.99	9	<0.0001
	Puerto Bolívar Boca	625.44	9	<0.0001
Salinity				
	Boca de Macho	837.16	9	<0.0001
	Héroes de Jambelí	230.17	8	<0.0001
	Isla de Jambelí	671.41	9	<0.0001
	Puerto Bolívar Adentro	464.85	9	<0.0001
	Puerto Bolívar Boca	619.21	9	<0.0001
pH				
	Boca de Macho	534.3	9	<0.0001
	Héroes de Jambelí	431.66	8	<0.0001
	Isla de Jambelí	245.91	9	<0.0001
	Puerto Bolívar Adentro	378.53	9	<0.0001
	Puerto Bolívar Boca	416.76	9	<0.0001
BGA				
	Boca de Macho	650.84	9	<0.0001
	Héroes de Jambelí	309.2	8	<0.0001
	Isla de Jambelí	469.75	9	<0.0001
	Puerto Bolívar Adentro	219.78	9	<0.0001
	Puerto Bolívar Boca	519.81	9	<0.0001

DF = degrees of freedom.

**Table 3:** Fixed effects coefficient statistics for associations between prevalence of *Vibrio cholerae* and each strain separately, and environmental variables, when controlling for site effects.

Environmental Variable	Prevalence	Estimate (SE)	z	*p*-Value
Temperature	*V. cholerae*	−0.60 (0.19)	−3.20	0.001
	Strain O1	−0.21 (0.22)	−0.93	0.35
	Strain O139	−0.68 (0.19)	−3.67	<0.001
Salinity	*V. cholerae*	−0.13 (0.06)	−2.27	0.02
	Strain O1	−0.10 (0.07)	−1.47	0.14
	Strain O139	−0.09 (0.06)	−1.45	0.15
pH	*V. cholerae*	3.45 (1.33)	2.60	0.01
	Strain O1	1.14 (1.65)	0.68	0.49
	Strain O139	3.86 (1.35)	2.86	0.004
BGA	*V. cholerae*	0.27 (0.06)	4.24	<0.0001
	Strain O1	0.005 (0.07)	0.07	0.94
	Strain O139	0.23 (0.06)	3.70	<0.0001
